# The Mental Health Status and Intellectual Ability of Unwed Mothers Dwelling in Korean Shelter Homes

**DOI:** 10.3390/ijerph15040637

**Published:** 2018-03-30

**Authors:** Suyeon Jo, Soowon Park, Jung Hae Youn, Bo Kyung Sohn, Hyo Jung Choi, Ji Yeon Lee, Jin Yong Lee, Jun-Young Lee

**Affiliations:** 1 Department of Psychiatry, SMG-SNU Boramae Medical Center, Seoul National University College of Medicine, Seoul 07061, Korea; suyeon.jo@daum.net (S.J.); jung830313@hanmail.net (H.J.C.); pavv_@naver.com (J.Y.L.); 2 Department of Education, Sejong University, Seoul 05006, Korea; swpark@emocog.com; 3 Department of Art Therapy & Counseling Psychology, Cha University, Gyeonggi-do 11160, Korea; jung_et@naver.com; 4 Department of Psychiatry, Inje University Sanggye Paik Hospital, Seoul 01757, Korea; bksohn1221@daum.net; 5 Public Health Medical Service, SMG-SNU Boramae Medical Center, Seoul National University College of Medicine, Seoul 07061, Korea

**Keywords:** unwed mother, mental health, intellectual ability, Korea

## Abstract

Although many unwed mothers have issues concerning mental health and intellectual ability, little research has focused on their mental and cognitive status. Due to the public stigma attached to unwed mothers in South Korea, they tend to conceal their status and are less likely to seek psychiatric and psychological help. In this context, this study aims to assess the current status of their mental health and intellectual characteristics. A total of 48 unwed mothers from two shelter homes in South Korea agreed to participate in the study. We compared the mental health status of these unwed mothers with that of the general female population. Unwed mothers were more likely than those of the general female population to have mood disorders, post traumatic stress disorder (PTSD), alcohol and nicotine use disorders, and attention-deficit hyperactivity disorder (ADHD). Among the 48 unwed mothers, 20 (41.7%) had an IQ of less than 70, and the mean IQ (78.31) was significantly lower than the normalized mean IQ of the general female population. This study confirmed that unwed mothers dwelling in Korean shelter homes are more likely than the general female population to have mental disorders.

## 1. Introduction

Out-of-wedlock birth rates have increased in Korea [1]. Based on statistical data from the Population and Housing Census, the number of unmarried mothers is estimated at about 13,000 and is increasing [2]. It is difficult to estimate the number of unwed mothers in Korea, considering the fact that becoming pregnant out of wedlock is considered shameful and undesirable [3,4]. Due to the public stigma attached to unwed mothers in Asian countries, these women tend to conceal their status and are less likely to seek proper support [5,6,7]. Due to the many burdens of unwed mothers, many unwed mothers in Korea decide to put up their children for adoption. However, Korea has seen a steady increase in child-rearing unwed mothers. According to a report from the Korean Women Institution, the proportion of unwed mothers who rear their children has increased from 5.8% in 1984 to 11% in 2001 and to 31.7% in 2005 [8]. Along with the recent increase in the number of single mothers, they tend to decide that staying in shelter homes is the safest option for child-rearing. Researches on unwed mothers who have stayed in the shelter homes may shed light on understanding their status and establishing policies for them.

Recent research suggests that many unwed mothers face many difficulties, including economic deprivation, discrimination, and biased stereotypes [3,4,9]. Moreover, they experience a wide range of psychiatric issues, such as excessive stress and depression, and psychological problems, such as social isolation and a lack of emotional support from family [5,10,11]. Little research has focused on the mental health and cognitive ability of unwed mothers. Some researchers have explored the relationships between the psychiatric disorders and intellectual disabilities of unwed mothers [12,13]. Experiencing mental disorders of unwed mothers may take a toll on their children, as well as themselves [14,15]. This can aggravate the status of one’s mental health. Furthermore, intellectual ability, use of alcohol and nicotine, and anti-social behavior can all affect the onset of early motherhood [13,16]. 

Unfortunately, the majority of unwed mothers in Korea are still suffering from economic difficulties and social prejudice despite the increase in government support and public awareness [1,8,9]. Based on the fact that social policies regarding unwed mothers has not fully supported the burden of unwed motherhood, comprehensive social welfare policies are essential to handle these problems that unwed mothers encounter in their child-rearing efforts [9]. Therefore, the mental health and intellectual ability of unwed mothers needs to be investigated. 

As of now, few studies on the psychiatric characteristics and intellectual abilities of unwed mothers in Korea exist. Investigation of characteristics of unwed mothers is essential to develop supportive programs and establish policies. In this context, this study aims to investigate the mental health and intellectual ability of unwed mothers, compared with the general female population in Korea.

## 2. Materials and Methods 

### 2.1. Participants and Data Collection 

Attempting to eliminate a medical and welfare blind spot, department of Psychiatry and department of Public Health Medical Service in SMG-SNU Boramae Medical Center executed a mental health assessment project of single mothers living in Korean shelter homes. Participants were recruited from two shelter homes, one of which has six branches. Among the 60 unwed mothers in these shelters, 48 unwed mothers agreed to participate in the study. Demographical information is presented in the Results section. All participants gave their informed consent for inclusion before they participated in the study. The study was conducted in accordance with the Declaration of Helsinki, and the protocol was approved by the Ethics Committee of SMG-SNU Boramae Medical Center (IRB No. 30-2017-19).

### 2.2. The General Female Population

Reference data were derived from a representative sample of the Korean adult population, consisting of 6022 participants. The participants were selected using a multi-stage cluster sampling method [17]. Trained interviewers accomplished face-to-face interviews with each of the participants in their homes from 25 March 2011 to 23 December 2011. As the study aim was to identity the difference between unwed women and women generally, the final sample was composed of 3714 women. More detailed information about this population and the data collection has been described in a previous study [18]. 

### 2.3. Measures

#### 2.3.1. The Structured Clinical Interview for DSM-IV (SCID) 

The Structured Clinical Interview for DSM-IV(SCID) was used to determine the mental health status of unwed mothers [19]. The Korean version of SCID has been validated [20]. Presence of psychiatric disorders, such as current and lifetime mood disorders (major depression disorder, dysthymia, and bipolar disorder), post-traumatic stress disorder (PTSD), alcohol use disorder, and nicotine use disorder, was recorded. Obligatory questions, criteria from the American Psychiatric Association’s DSM-IV, a classification of rating symptoms, and an algorithm for final diagnosis were administered. The SCID allows interviewers to use all information to determine the absence or presence of symptoms. 

#### 2.3.2. Adult ADHD Self-Report Scale (ASRS-v1.1)

The ASRS was developed to screen attention-deficit hyperactivity disorder (ADHD) in adults in conjunction with all 18 DSM-IV Criterion A symptoms of adult ADHD [21]. The full ASRS is an 18-item self-report questionnaire. Of 18 questions, six questions were the most predictive of ADHD symptoms [22]. In this study, six items were used to confirm adults suffering from the symptoms of ADHD validated for use [23]. The participants who checked four or more marks within Part A were regarded as having ADHD symptoms.

#### 2.3.3. Korean Wechsler Adult Intelligence Scale-IV (K-WAIS-IV) Short Forms

The intellectual ability was examined using Korean Wechsler Adult Intelligence Scale-IV (K-WAIS-IV) Short Forms, which was based on the criteria in Wechsler Adult Intelligence-IV [24]. The form consisted of four sections: information, matrix reasoning, arithmetic, and symbol search. The short form was validated by Choi et al. [25]. The cutoff IQ score in this study is 70 [26].

### 2.4. Procedures 

The 48 unwed mothers were recruited from two shelter homes of South Korea from September 2016 to December 2016. Two research clinicians underwent training sessions for administrating the SCID, K-WAIS-IV. The average administration time was 90 min. The protocol of the present study was approved by the review board at SMG-SNU Boramae Medical Center in Korea, and all procedures were completed in accordance with the guidelines of the Helsinki Declaration. 

### 2.5. Statistical Analysis

We analyzed descriptive statistics (mean, SD, and frequencies) to examine the characteristics of demographic variables (i.e., age, education, type of health insurance, employment, and experience of mental illness), psychiatric disorders as indicators of mental health status (mood disorders, PTSD, alcohol use, nicotine use, and ADHD), and intellectual ability. To assess the prevalence of psychiatric disorders, two sample *t*-tests were conducted between 48 unwed participants and 3714 general participants. The data were analyzed by logistic regression to examine the extent to which association between psychiatric disorders and binary variables (unwed and general female population). To explore the variables directly associated with psychiatric disorders, we used multiple logistic analysis. In this analysis, dependent variables were calculated by the sum of present and lifetime prevalence of the respective disorders (mood disorders, PTSD, alcohol use, nicotine use, and ADHD). Of the sociodemographic variables, age, education year, and type of health insurance, which are known to affect mental illness, were included for analysis. We also performed multiple logistic regression to assess factors associated with psychiatric disorder in unwed mothers. Age and education year were included as continuous variables. Other variables were regarded as binary variables. Lastly, a sample *t*-test was conducted to examine whether the unwed participants’ intellectual ability was statistically different from a randomized population mean. To do this, we generated randomized data based on IQ scores that show a population mean of 100 and a standard deviation of 15. This was done because information of intellectual ability was not included in the reference data. All analyses were completed using SPSS for Windows 18.0 (SPSS, Inc.; Chicago, IL, USA). All statistical tests were two-sided and a *p*-value < 0.05 was considered statistically significant. 

## 3. Results

### 3.1. Sociodemographic Characteristics

Sociodemographic information including age, years of education, types of medical insurance, states of employment, and experience of mental illness of 48 Korean unwed mothers and 3714 women from the general population are presented in Table 1. The mean age of unwed mothers was 21.77 (*SD* = 6.50) and the mean years of education was 11.22 (*SD* = 2.30). The mean age of the general female population was 48.29 (*SD* = 15.37) and the mean years of education was 11.02 (*SD* = 6.27). 

### 3.2. Mental Health Status

Table 2 shows the mental health problems (i.e., mood disorders, PTSD, alcohol use disorders, nicotine use disorders, and ADHD) of unwed mothers and the general female population. The results provide empirical evidence that unwed mothers experience psychiatric disorders more often than the normal population. In the case of mood disorders, major depressive symptoms (12.5%), dysthymia (6.3%), and lifetime bipolar disorder (12.5%) were significantly more prevalent in unwed mothers than for in the general female population. In terms of psychiatric disorders, lifetime PTSD (37.5%), lifetime alcohol use disorder (50.0%), and lifetime alcohol use disorder (41.7%) were significantly different in unwed mothers compared with the general female population. Moreover, unwed mothers (12.5%) were substantially more likely to have attention problems than other people.

Moreover, Table 2 presents the results of logistic regression to estimate odds ratios. Compared with the general female population, unwed mothers are more likely to experience psychiatric disorders, except for present PTSD and present alcohol use disorder. Of the psychiatric disorders, the prevalence of lifetime nicotine use disorder (48.31 times) was the highest, followed by lifetime PTSD, ADHD, lifetime alcohol, present nicotine, lifetime mood disorder, present PTSD, present mood disorder, and present alcohol. 

Table 3 shows the results of an analysis of the association between sociodemographic characteristics and psychiatric disorders. Unwed mothers have significantly higher odds of having mental disorders compared with the general female population. PTSD (25.88 times), nicotine use (9.72 times), and mood disorder (9.61 times) in unwed mothers were higher than other psychiatric disorders. Furthermore, age and education year were significant predictors of mental disorder. Moreover, a shorter education year is negatively associated with mood disorder, PTSD, and ADHD. Lastly, women who have more stable job status and health insurance are more likely to affect alcohol and nicotine use disorder.

To define the factors associated with the psychiatric disorder of unwed mothers, we conducted logistic regression analysis to adjust for the effect of variables. In this model, sociodemographic variables, age and education year, intelligence quotient, type of health insurance, and employment were included, considering previous research results. Age and education year is significantly associated with single motherhood [27,28]. Moreover, the type of insurance and employment are indicators of economic status [29,30]. Even though there are no significant predictors in the model, slightly similar trends were found in age and type of health insurance (Table 4).

### 3.3. Intelligent Quotient (IQ)

**Figure 1:**
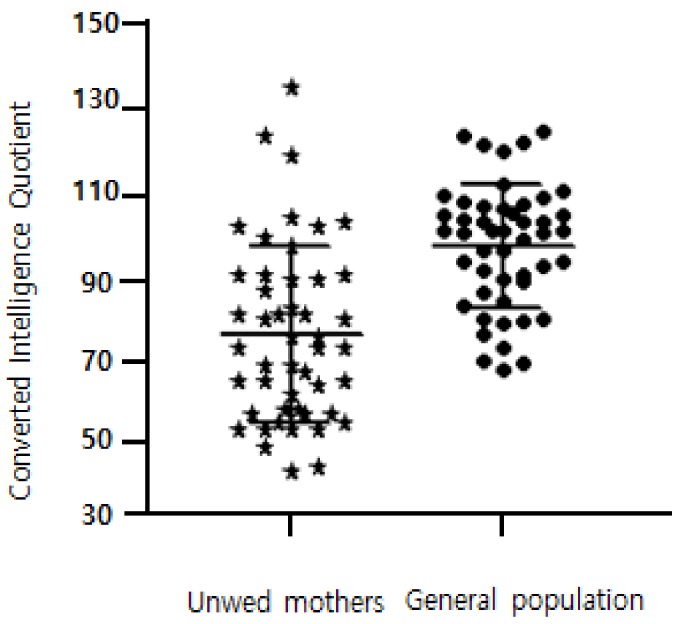
Difference in distribution of converted IQ scores between 48 unwed mothers and 48 of randomly generated population.

An IQ of 70 or lower constitutes a learning disability [26,31]. Figure 1 showed the differences in distribution of IQ between two groups. Among 48 participants, 20 (41.7%) had an IQ of less than 70 and the mean IQ (78.31) was significantly lower (*t* (47) = 6.94, *p* < 0.05) than the normalized mean IQ (100) of the general female population. 

## 4. Discussion

The study focused on assessing current mental status of unwed mothers. Taken as a whole, the findings in this study indicate that there is a significant difference in psychiatric characteristics between unwed mothers and the general female population. This is, to our knowledge, the first study to evaluate unwed mothers’ mental health and intellectual problems in South Korea.

The proportion of unwed mothers with chronic disorders was significantly higher than that of the general female population. Our data indicate that unwed mothers are 28.32 times more likely to have experienced traumatic events than the general female population. Unwed mothers reported that they experienced sexual assault (12.5%), physical violence (14.6%), social bullying (4.2%), and suicide (4.2%). These results are consistent with prior research that sexual and physical abuse have been related to detrimental outcomes such as risky sexual behavior, early pregnancy, and mental illness [31,32,33,34,35]. However, some studies suggest that sexual attitudes of peer groups are more important indicators of pregnancy than sexual assault experiences [36,37]. Though these results are in conflict with our findings, our study provides empirical evidence that women who have experienced traumatic events are at greater risk of early pregnancy related with being unwed mother. Our results also indicates that women with less education are more vulnerable to post-traumatic stress. 

Unwed mothers are more likely to experience more mood disorders than the general female population. Unwed mothers reported suffering from depression (50%), dysthymia (6.3%), and bipolar disorder (12.5%) at some point in their lifetime. Unwed mothers who stayed in shelters are more likely to have non-supportive relationships with their family compared to the general female population [2,4]. This is in line with studies demonstrating that unwed mothers reported significantly high rates of depressive symptoms [5,10,38]. Many adolescent mothers have been shown to have significantly severe depression, prenatally and after birth [15,39]. Stress originates from parenting, and economic hardship may accelerate the level of depression [40]. In this respect, the process underlying the association between single motherhood and depression should be further explored. In addition, employment status was negatively related to mood disorder. This might mean that people with mood disorders are not likely to find work. This is consistent with previous studies showing that it is difficult to perform economic activities without physical and social support [29,41].

In Korean society, single mothers can face numerous difficulties. Some studies have pointed out that many single mothers suffer from social condemnation, having difficulty with basic living expenses, being discriminated in the workplace, and so on [9,42]. The general female population does not suffer from these issues to the same extent, and adolescent mothers may suffer from these issues to a greater extent. Adolescent mothers sometimes stay in residential facilities, as they are not prepared to live alone [4,8,42]. Because of the social stigma, they are reluctant to seek adequate care when they are pregnant [3,7]. Considering the fact that unwed mothers are at even greater risk [3,37], it is essential for health authorities to particularly consider the plight of unwed mothers. 

Our findings indicate that unwed mothers, compared to the general female population, are more exposed to alcohol and nicotine use in their lifetime. Staying in residential facilities that prevent mothers from drinking and smoking does not necessarily decrease this risk. These findings are in agreement with a study showing that unwed mothers are more likely to consume alcohol and nicotine [43,44,45]. Prior studies suggest that frequent use of drug use and smoking is strongly related to early pregnancy, since habitual use is a sign of having difficulties in their life [13,16,46]. Moreover, our data show that unwed mothers tend to have more attention problems than the general female population. This finding corroborates past research showing that single motherhood is associated with attention-deficit hyperactivity disorder (ADHD) [47,48].

In this study, unwed mothers had less intellectual ability than the general population. This result is consistent with previous studies showing that certain psychiatric disorders are associated with intellectual ability [49,50,51]. Some research indicates that bipolar disorder is significantly associated with intellectual inefficiency [52,53]. However, conflicting evidence has been provided in prior research showing that other factors, such as prenatal care, family support, and other environmental factors, influence mental health [3,10,29,47]. Further research is needed to illuminate the causal relationship between the psychiatric disorders and intellectual ability of unwed mothers and of the general population.

Our findings imply that Korean health authorities need to act with respect to unwed mothers. The policy debate concerning universal insurance coverage needs to takes this into consideration. Based on our findings, health authorities of interventions aimed at supporting unwed mothers should consider mental health treatment when formulating policy. Furthermore, mental health professionals treating unwed mothers need to be sensitive to their higher risk of psychiatric disorders, while community-based authorities also need to be aware of the mental state of single mothers. Although educational attainment is not sufficient to improve mental health [40,54,55], these results could be interpreted as suggesting that reorganized policies and programs are needed. It appears that providing comprehensive prevention programs would be helpful in reducing the status of mental health over the long term.

This study has several limitations. First, the study is limited by selection bias. Our data collection was conducted on a small sample of women from shelter facilities and does not represent all unwed mothers in Korea. One possible reason for this shortcoming is that, considering the public stigma attached to being an unwed mother, it is difficult for unwed mothers to openly participate in such a study. In addition, to represent the general female population, multi-stage cluster sampling was used in the reference data. More ideal sampling is needed to utilize a nationally representative sample. In case of generated data, as intelligence ability information was not included in the data, we generated a randomized population to assess. In this respect, further study should involve a nationally representative survey of intelligence ability of Korean adults. Furthermore, the results of this study do not benefit from directionality or causality. While our findings suggest that unwed mothers experience mental disorders significantly more often than the general female population does, there may be differences in physical conditions. Our study does not consider environmental factors affecting unwed mothers, such as social support and economical support from the government. In future studies, conditions affecting mental health and intellectual ability should be investigated. Lastly, longitudinal studies would be able to yield a more comprehensive picture of how individual and environmental factors influence unwed mothers and their children. 

In spite of its limitations, our study confirms that Korean unwed mothers tend to suffer from mental disorders more often than the general female population. This study suggests that potential intervention should be designed to eliminate the stigma and social discrimination of unwed mothers. These findings will be valuable for informing policy makers and shelter workers who care with unwed mothers.

## 5. Conclusions

This study confirms that unwed mothers dwelling in Korean shelter homes are more likely than the general female population to suffer from mental disorders. Therefore, supportive programs should be developed or policies established to improve the quality of life of unwed mothers in Korea.

## Figures and Tables

**Table 1:** Sociodemographic variables of unwed mothers (*N* = 48) and the general female population (*N* = 3714).

*N* (%)		
Variables	Unwed Mothers	General Female Population
Age (years)		
≤17	12 (25)	0 (0)
18–29	32 (66.7)	457 (12.3)
30–39	1 (2.1)	772 (20.8)
40–49	3 (6.3)	740 (19.9)
50–59	0 (0)	699 (18.8)
60–69	0 (0)	636 (17.1)
70–74	0 (0)	410 (11.0)
Education		
<Elementary school	0 (0)	208 (5.6)
Elementary school	0 (0)	658 (17.7)
Middle school	10 (20.8)	449 (12.1)
High school	30 (62.5)	1195 (32.2)
University	8 (16.7)	1204 (32.4)
Type of health insurance		
National Health Insurance (NHI)	19 (39.6)	3419 (92.1)
Medical Aid (MA)	29 (60.4)	111 (3.0)
Employment		
Employed	30 (62.5)	1067 (28.7)
Part time	0 (0)	234 (6.3)
Unemployed	18 (37.5)	2408 (64.8)
Experience of mental illness		
Yes	10 (20.8)	373 (10)
No	38 (79.2)	3092 (83.3)

Note. *N* values do not always total 48 (unwed mothers) and 3714 (general female population) due to missing responses on some variables.

**Table 2:** Mental health status of unwed mothers and the general female population.

Symptom	*N* (%)		*t*	OR(95% CI)
	Unwed Mothers (*N* = 48)	General Female Population (*N* = 3714)		
Mood Disorder				
Present	7 (14.6)	154 (4.1)	3.59 *	5.05 (2.24–11.39) *
Lifetime	26 (54.2)	374 (9.9)	9.96 *	10.54 (5.92–18.79) *
PTSD				
Present	2 (4.2)	28 (0.8)	2.56	5.72 (1.32–24.74)
Lifetime	18 (37.5)	77 (2.0)	15.45 *	28.32 (15.14–52.98) *
Alcohol use				
Present	2 (4.2)	39 (1.1)	2.01	4.10 (0.96–17.47)
Lifetime	24 (50.0)	183 (4.9)	13.65 *	19.24 (10.72–34.54) *
Nicotine use				
Present	4 (8.3)	28 (0.8)	5.48 *	11.97 (4.03–35.56) *
Lifetime	20 (41.7)	54 (1.4)	20.24 *	48.31(25.64–91.03) *
ADHD				
Present	6 (12.5)	25 (0.7)	8.85 *	21.48 (8.37–55.13) *
Lifetime	-	-	-	-

Note: * *p* < 0.05; PTSD: post-traumatic stress disorder; ADHD: attention-deficit hyperactivity disorder; OR: odds ratio; *N* values do not always total 48 (unwed mothers) and 3714 (general female population) due to missing responses on some variables.

**Table 3:** ORs from multiple logistic regression analysis of the probability of psychiatric disorder.

Variables	aOR (95% CI)				
	Mood Disorder	PTSD	Alcohol Use	Nicotine Use	ADHD
Group					
General Female Population	1.00	1.00	1.00	1.00	1.00
Unwed mothers	9.61 (5.19–17.82) *	25.88 (12.34–54.26) *	4.93 (2.69–9.02) *	9.72 (5.14–18.38) *	10.25 (3.30–31.83) *
Age	0.86 (0.79–0.93) *	0.88 (0.74–1.04)	0.82 (0.76–0.89)*	0.98 (0.89–1.08)	0.80 (0.62–1.04)
Education year	0.76 (0.68–0.84) *	0.80 (0.65–0.99) *	1.05 (0.95–1.17)	1.06 (0.93–1.20)	0.58 (0.42–0.79) *
Type of health insurance					
NHI	1.00	1.00	1.00	1.00	1.00
MA	1.16 (0.97–1.39)	1.27 (0.92–1.75)	1.21 (1.01–1.45) *	1.24 (1.00–1.54) *	1.39 (0.91–2.13)
Employment					
Employed	1.00	1.00	1.00	1.00	1.00
Unemployed	0.76 (0.64–0.89) *	0.84 (0.60–1.16)	1.60 (1.40–1.82) *	1.65 (1.39–1.94) *	1.34 (0.86–2.08)

Note: * *p* < 0.05; aOR: adjusted odds ratio; PTSD: post-traumatic stress disorder; ADHD: attention-deficit hyperactivity disorder. NHI: national health insurance; MA: medical aid.

**Table 4:** Factors associated with psychiatric disorders in unwed mothers.

Variables	aOR (95% CI)	*p* Value
Age	0.92 (0.71–1.19)	0.523
Education year	2.98 (0.64–13.91)	0.165
Intelligent Quotient (IQ)	0.82 (0.67–1.01)	0.057
Type of health insurance		
National Health Insurance(NHI)	1.00	
Medical Aid(MA)	2.96 (0.16–56.67)	0.471
Employment		
Employed	1.00	
Unemployed	0.04 (0.01–1.33)	0.071

Note: aOR, adjusted Odds Ratio.
